# Baseline Objective Malnutritional Indices as Immune-Nutritional Predictors of Long-Term Recurrence in Patients with Acute Ischemic Stroke

**DOI:** 10.3390/nu14071337

**Published:** 2022-03-23

**Authors:** Xiaoyan Han, Jinhua Cai, Youjia Li, Xiaoming Rong, Yi Li, Lei He, Honghong Li, Yuchan Liang, Huiqin Huang, Yongteng Xu, Qingyu Shen, Yamei Tang

**Affiliations:** 1 Department of Neurology, Sun Yat-sen Memorial Hospital, Sun Yat-sen University, Guangzhou 510120, China; hanxy28@mail2.sysu.edu.cn (X.H.); cjhua@mail2.sysu.edu.cn (J.C.); rongxm@mail.sysu.edu.cn (X.R.); eleam2002@163.com (Y.L.); helei33@mail.sysu.edu.cn (L.H.); leehh88@163.com (H.L.); xuyt1990@163.com (Y.X.); 2 Department of Neurology, First People’s Hospital of Zhaoqing, Zhaoqing 526000, China; lyj2102353@163.com (Y.L.); ycliang1986@163.com (Y.L.); hhq186699@163.com (H.H.); 3 Guangdong Provincial Key Laboratory of Malignant Tumor Epigenetics and Gene Regulation, Sun Yat-sen Memorial Hospital, Sun Yat-sen University, Guangzhou 510120, China; 4 Guangdong Province Key Laboratory of Brain Function and Disease, Zhongshan School of Medicine, Sun Yat-sen University, Guangzhou 510120, China

**Keywords:** malnutrition, immunity, acute ischemic stroke, recurrent ischemic stroke, long-term prognosis, the controlling nutritional status score, the prognostic nutritional index score

## Abstract

Background: The controlling nutritional status (CONUT) score and the prognostic nutritional index (PNI) score were designed as indicators of patients’ immune-nutritional status. This study aimed to investigate the prognostic impact of the CONUT and PNI scores on long-term recurrent ischemic stroke (RIS) and adverse outcomes for adults with acute ischemic stroke (AIS). Methods: This retrospective study enrolled 991 AIS patients. Multivariable Cox regression models were used to assess the relationships of the malnutritional indices and RIS and major cardiovascular events (MACEs). Results: During a median follow-up at 44 months (IQR 39–49 months), 203 (19.2%) patients had RIS and 261 (26.3%) had MACEs. Compared with normal nutritional status, moderate to severe malnutrition was significantly related to an increased risk of RIS in the CONUT score (adjusted hazard ratio (HR) 3.472, 95% confidence interval (CI) 2.223–5.432, *p <* 0.001). A higher PNI value tertile (tertile two, adjusted HR 0.295, 95% CI 0.202–0.430; tertile three, adjusted HR 0.445, 95% CI 0.308–0.632, all *p <* 0.001) was related to a lower risk of RIS. Similar results were found for MACEs. The PNI exhibited nonlinear association with the RIS and both two malnutritional indices improved the model’s discrimination when added to the model with other clinical risk factors. Conclusions: This study demonstrated that the CONUT and PNI are promising, straightforward screening indicators to identify AIS patients with impaired immune-nutritional status at higher risk of long-term RIS and MACEs.

## 1. Introduction

Stroke is the leading cause of disability and death among adults globally [1]. The majority of strokes are ischemic and survivors are still at increased risk for having a recurrence, which is often more severe and disabling than the index event [2]. Ischemic stroke secondary prevention remains a clinical challenge. Hence, strategies to reduce the burden of ischemic stroke are pressing. Early identification of potentially modifiable risk factors (e.g., obesity and hypertension) could offer an alternative approach to reduce a patient’s risk of recurrence.

Malnutrition is a common problem in the stroke population. The prevalence of premorbid malnutrition was around 33% to 34.3% for acute ischemic stroke (AIS) patients at admission [3,4]. Recent evidence demonstrated that malnutrition at admission was related to unfavorable stroke outcomes in AIS patients, such as increased mortality and poor neurological function at 90 days of onset [5,6,7]. The impact of malnutrition on prognosis after AIS exists not only in the acute stage but also in the chronic phase [7]. Thus, early screening of nutritional status at admission is critical in patients with AIS, allowing timely and effective nutritional intervention. Traditional malnutrition screening tools, such as the Nutritional Risk Screening 2002 and Malnutrition Universal Screening Tool, are exhaustive and time-consuming. Furthermore, the subjective part of these screening tools could be challenging to assess in stroke patients, owing to stroke-related neurological deficits. Therefore, objective and blood-based malnutritional indices may circumvent the limitations inherent to subjective malnutrition screening tools.

The controlling nutritional status (CONUT) score and the prognostic nutritional index (PNI) score, which can be calculated quickly from routine blood-based parameters based on total peripheral lymphocytes count, serum albumin, and total cholesterol, were originally designed to assess malnutritional and immunological risks in patients undergoing surgery [8,9]. Their prognostic significance on long-term mortality has been reported in patients with cardiovascular diseases [10,11]. Previous studies regarding the clinical implication of the two nutritional indicators in AIS have been mainly validated in predicting the short-term prognosis or in elder populations [3,12,13]. However, the utility of these screening tools in predicting long-term adverse outcomes after AIS in the general population remains unclear.

To address these gaps, the aim of the present study was to investigate the association of PNI and CONUT scores with long-term recurrent ischemic stroke (RIS) and adverse clinical outcomes in adults with AIS.

## 2. Materials and Methods

### 2.1. Study Population

This was a STROBE-compliant, single-center retrospective study involving patients with AIS admitted to the First People’s Hospital of Zhaoqing. Patients admitted to our hospital with a final diagnosis of AIS between January 2016 and June 2018 were enrolled. Patients were included if they were (a) aged ≥18 years old, (b) with a diagnosis of acute ischemic stroke within 7 days of onset, (c) with a new lesion on a diffusion-weighted imaging (DWI) sequence of the brain using magnetic resonance imaging (MRI) scan immediately prior to or during hospitalization, and (d) had completed at least three years of follow-up or had previously deceased. Patients were excluded in the presence of any of the following conditions: (a) incomplete medical records or missing data, (b) history of systemic inflammatory diseases, malignant tumor, hematological diseases, (c) history of severe hepatic, renal, or cardiac dysfunction, or (d) treatment with intravenous thrombolysis or endovascular therapy after admission.

### 2.2. Ethics Statement

This study was performed retrospectively using clinical records and in compliance with the Declaration of Helsinki for investigations involving humans. After clinical information was collected, patient identifiers were removed and subsequently, patients could not be identified either directly or indirectly. This retrospective, observational study was approved by the Ethics Committee of the First People’s Hospital of Zhaoqing (approval number: B2021-11-02), who decided that the need for signed informed consent was waived.

### 2.3. Demographic and Clinical Data

Demographic data and baseline medical history about age, sex, history of hypertension, history of diabetes mellitus, history of stroke, and smoking status were collected at admission. All blood indices were defined as the first test result within 24 h of admission. Stroke etiologies were categorized following the Trial of ORG 10172 in Acute Stroke Treatment (TOAST) classification [14]. The National Institutes of Health Stroke Scale (NIHSS) score was used to assess the severity of stroke [15]. The premorbid functional status was estimated using the modified Rankin Scale (mRS) score. Neurological deterioration was defined as an increase in the NIHSS score by ≥4 points during hospitalization [16].

### 2.4. Malnutrition Screening Tools

The CONUT score was estimated using serum albumin concentration, peripheral lymphocyte count, and the total cholesterol concentration. The PNI score was estimated utilizing the following formula: 5 × lymphocyte count (10^9^/L) + serum albumin concentration (g/L). The two nutritional scoring systems are described in Table 1 [9,17].

### 2.5. Clinical Outcomes

The clinical outcomes were derived from follow-up data of patients with acute ischemic stroke from a single comprehensive stroke center registry. Stroke recurrence, mortality, and acute coronary syndrome (ACS) were recorded during follow-up. The primary outcome was defined as the recurrence of ischemic stroke and the secondary outcome was the composite of major cardiovascular events (MACEs), including mortality, acute coronary syndrome, and stroke recurrence. Patients were followed up for outcomes after AIS onset. The study was censored on 1 August 2021.

### 2.6. Statistical Analysis

Statistical analyses data were presented as median (interquartile range, IQR) or as numbers and percentages (%). After testing the distribution of each parameter by the Shapiro–Wilk test, none of them were normally distributed. Therefore, the Mann–Whitney U test was used to compare the two groups involving continuous variables and the chi-square test or Fisher’s exact test, as appropriate, was used to compare the two groups involving categorical variables. The CONUT score was analyzed continuously and categorized into three groups (absent, mild, moderate-severe) because the number of patients in the severe class was too small for detailed analysis. The PNI score was evaluated continuously and categorically in tertile and the number of malnutrition cases (4%) was too little according to the PNI standard scoring system. The log-rank test was used to compare the Kaplan–Meier curves of different risk groups. The independent relationships between the malnutrition indices and RIS and MACEs in the present study were investigated by univariate and multivariable Cox proportional hazards regression analyses. Model 1 was adjusted for age and sex as confounding factors. Model 2 was adjusted for age and sex, adding stroke etiology, NIHSS score at admission, and common vascular risk factors at admission (smoking status and history of ischemic stroke, diabetes mellitus, and hypertension). For Model 3, with *p* < 0.05 threshold, we selected the variables associated with RIS with a univariate Cox proportional hazards regression analysis. The model selection was performed by a forward stepwise selection procedure apart from the hematologic indices which were included in CONUT and PNI score calculation. After applying the Schoenfeld residuals test to confirm the proportional hazards assumption, no relevant violations were discovered. To assess the incremental prognostic value of the two malnutritional indices in Model 3, we employed the net reclassification index (NRI) and integrated discrimination improvement (IDI) [18]. Subsequently, the benefits and improved performance of different models with or without the malnutrition indices were compared by using decision curve analysis (DCA) [19].

We further performed a Cox regression model with restricted cubic splines adjusted for the same covariates included in Model 3 to examine the significance and pattern of the two malnutrition indices in the association with RIS and MACEs [20,21]. To balance best fit and overfitting, restricted cubic splines were generated with 5 knots (at fifth, 27.5th, 50th, 72.5th, 95th percentiles) to examine the potential nonlinear associations between malnutrition indices and adverse outcomes. 

Subgroup analyses were performed according to age, sex, whether the first-ever stroke, and stroke subtype. The association of the two malnutritional indices with the primary outcome in each subgroup was assessed using a multivariable Cox regression model adjusted for the covariates included in Model 3. All analyses were performed using R for Windows (version 4.0.5, R Foundation, Vienna, Austria), and statistical significance was set at a *p*-value less than 0.05.

## 3. Results

### 3.1. Clinical Characteristics and Prevalence of Malnutrition

**Figure 1:**
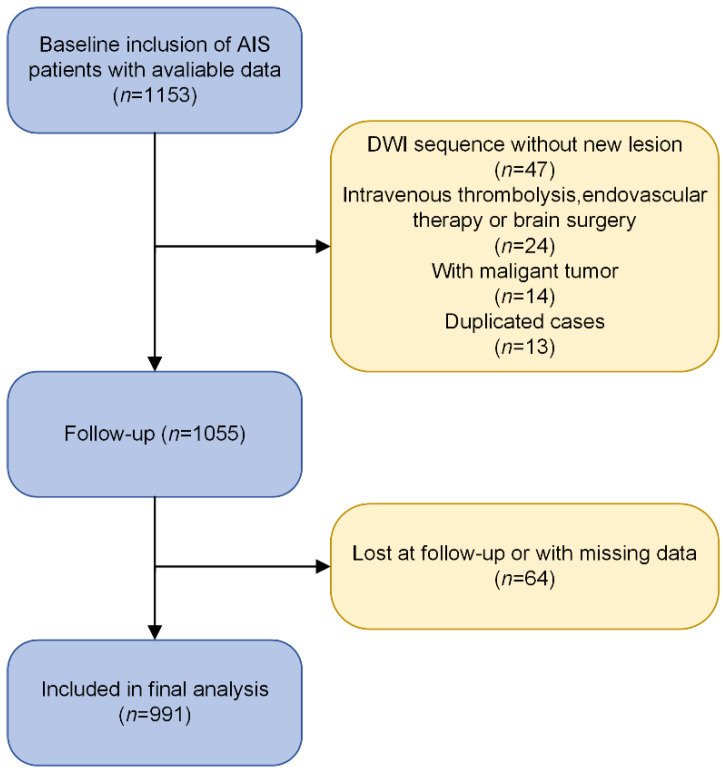
The flow chart of the current study protocol is shown. Abbreviations: DWI, diffusion-weighted image.

Among the 1153 participants at baseline, a total of 991 (85.9%) were eligible for the final analysis. 98 (8.5%) individuals were excluded because of violating our eligibility criteria at baseline, and 64 (5.5%) individuals were lost at follow-up or with missing data. The flow chart of the current study protocol is described in Figure 1. The median age of the participants was 66 (58, 74) years, 699 (71.1%) were male, the median of NIHSS scores at admission was3 (2, 5), the median CONUT score was 2 (0, 3), and the median PNI score was 46.3 (43.55, 50.15). According to the PNI and CONUT standard scoring systems, the percentage of AIS patients with combined malnutrition differed from 4% to 51%, respectively. The baseline demographic and clinical characteristics are detailed in Table 2. In brief, patients with RIS were older, with a higher proportion of hypertension and history of ischemic stroke, higher serum creatinine (Scr) levels, and lower serum albumin and total lymphocyte levels than patients without RIS (all *p* < 0.05).

### 3.2. Malnutrition Scores and Adverse Clinical Outcomes

**Figure 2:**
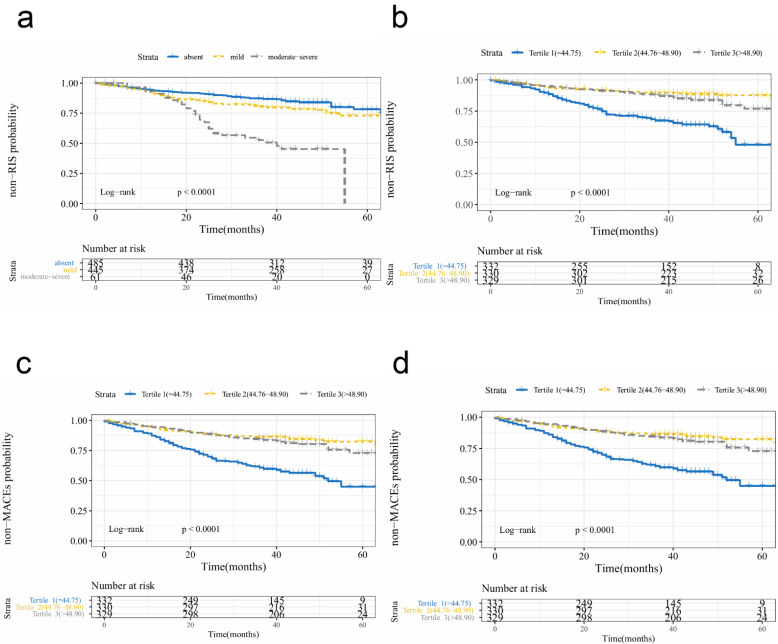
Kaplan–Meier survival curves of recurrent ischemic stroke by CONUT (**a**) and PNI (**b**); Kaplan–Meier survival curves of MACEs by CONUT (**c**) and PNI (**d**). Abbreviations: CONUT, Controlling Nutritional Status score; PNI, Prognostic Nutritional Index; RIS, recurrent ischemic stroke; MACE, major cardiovascular events.

During a median follow-up at 44 (39–49) months, 203 (19.2%) patients had ischemic stroke recurrence, 29 (2.9%) had incidental intracerebral hemorrhage, 58 (5.8%) died, and 261 (26.3%) had MACEs. Factors associated with RIS were examined using the univariate Cox regression analysis (Supplementary Table S1). The univariate predictors of RIS were age, history of hypertension, serum creatinine, lymphocyte count, platelet count, albumin level, stroke etiology, and neurological deterioration. We found that worsening nutritional status was related to a higher incidence of adverse clinical outcomes (RIS and MACEs), irrespective of the malnutritional indices when treated as continuous variables (Supplementary Table S1) or categorical variables (Figure 2).

Table 3 shows the relationship between the scores of the two malnutritional indices and RIS. In multivariable analysis, malnutrition (moderate-severe risk versus absent risk) was related to a significant increase in RIS risk using CONUT (adjusted hazard ratio (HR), 3.472; 95% confidence interval (95% CI), 2.223–5.423; *p* < 0.001) (Table 2, Model 3). After categorization of PNI into tertiles, we identified that those in the higher tertile groups of PNI scores were more likely to have a decreased risk of RIS than participants with the lowest PNI tertile groups in multivariable analysis adjusted covariates of Model 3 (adjusted HR: 0.295 (95 CI%: 0.202–0.430), 0.445 (95% CI: 0.308–0.632) for tertile two and tertile three, respectively, with all *p <* 0.001]. The significant association of the two malnutritional indices persisted when CONUT and PNI were analyzed as a continuous variate in Model 3 (all *p* < 0.001). The observed association of the two malnutritional indices with MACEs remained significant in multivariable analysis (Supplementary Table S2).

**Figure 3:**
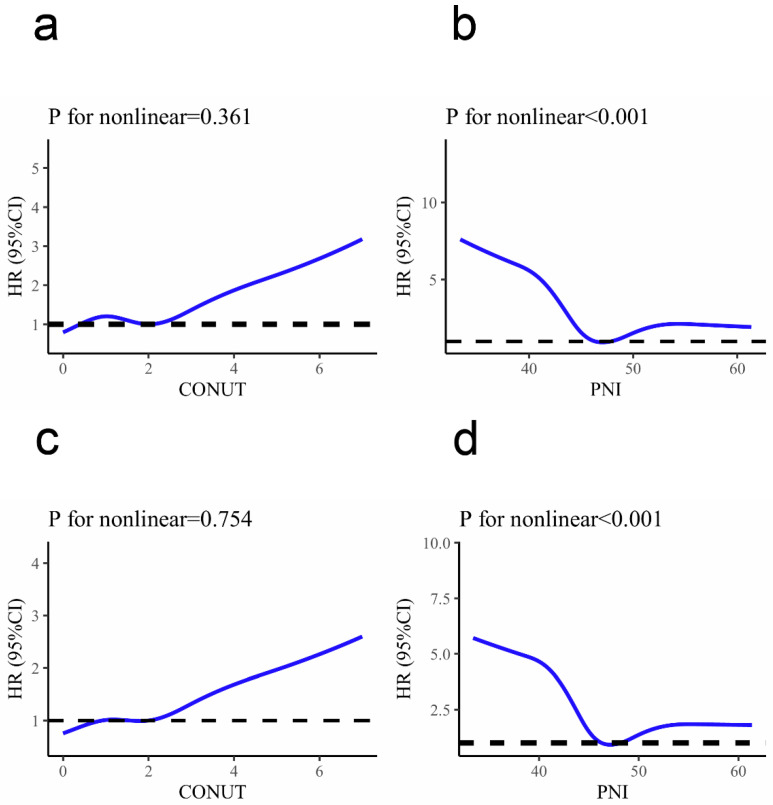
Association of CONUT (**a**) and PNI (**b**) with recurrent ischemic stroke, and association of CONUT (**c**) and PNI (**d**) with MACEs were fitted with restricted cubic spline with five knots (at fifth, 27.5th, 50th, 72.5th, and 95th percentiles), adjusted for covariates included in Model three in Table 3. The solid line represents the hazard ratio and the purple lines represent the 95% confidence interval. Abbreviations: RIS, recurrent ischemic stroke; MACEs, major cardiovascular events; CONUT, controlling nutritional status score; PNI, prognostic nutritional index score.

Restricted cubic splines (RCS) were used to assess the non-linear association of CONUT and PNI scores with the adverse clinical event (Figure 3). Regarding the U-shaped association of PNI and RIS, the plot indicated a substantial decrease in risk initially, reaching a minimum risk around 46.18, and an increase and relative flattening of risk thereafter (*p* for non-linearity < 0.001). A similar U-shaped relationship between PNI and MACEs was observed. The CONUT score demonstrated a linear association with clinical events. The risk was relatively flat for CONUT scores below two and increased rapidly thereafter (*p* for non-linearity > 0.05).

**Figure 4:**
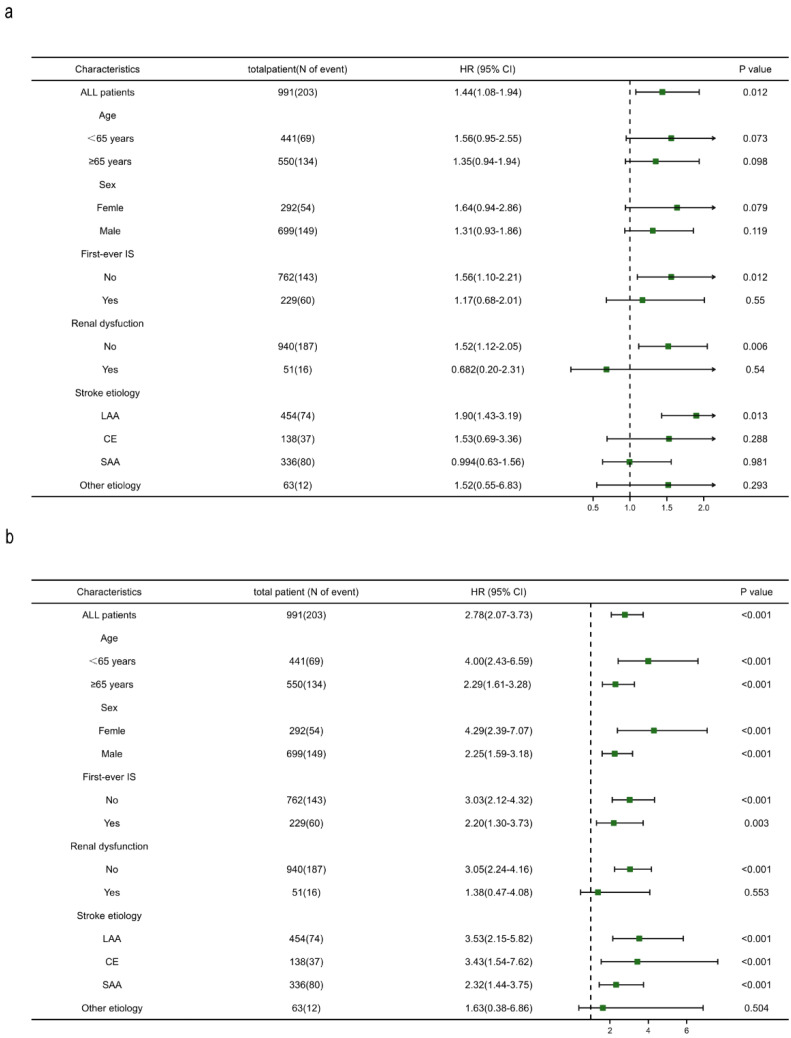
Cox regression analysis adjusted for covariates included in Model 3 in Table 3 were used to examine the association of the malnutritional index (CONUT > 1 versus CONUT ≤ 1, (**a**); PNI ≤ 44.75 versus PNI > 44.75), (**b**) among different subgroups.

In subgroup analysis, undernutrition defined as PNI ≤ 44.75, remained associated with RIS, except in the subgroup of patients with other etiologies of stroke. The associations between malnutrition according to CONUT (CONUT > 1) and RIS were not significant for age or gender subgroups, while different patterns were observed in the first-ever stroke and different stroke subtypes (Figure 4). When compared with the normal nutrition group, the adjusted hazard ratios of the malnutrition group (CONUT > 1) were 1.56 (1.10–2.21) for RIS (*p* = 0.012) for non-first-ever stroke patients and 1.90 (1.43–3.19) for RIS (*p* = 0.013) for large-artery atherosclerosis stroke patients, while no significant differences were detected for first-ever stroke patients and patients with other stroke subtypes.

### 3.3. Incremental Prognostic Value of Malnutritional Index for RIS

**Figure 5:**
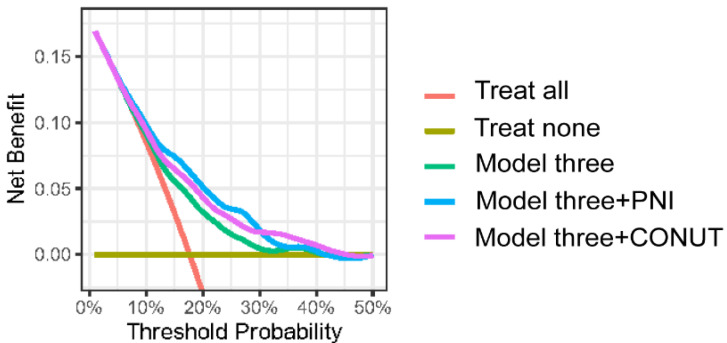
Decision curve analysis for Model three integrating PNI/CONUT features compared with Model three without using the malnutritional index. The blue line representing the model integrating PNI and the purple line representing the model integrating CONUT lie above the other models.

Through computation of net reclassification improvement (NRI) and integrated discrimination improvement (IDI) indices, we emphasized the additional value of malnutritional indices to classify patients according to RIS and MACEs when taken together with traditional risk factors included in Model 3, as indicated by the positive NRI and IDI coefficients in all models (Table 4). Additionally, decision curve analysis for the three models at three years is presented in Figure 5. The decision curve demonstrates that using the combination of PNI or CONUT features to predict RIS adds more net benefit than using the clinical features included in Model three alone.

## 4. Discussion

In this study, we identified a significant association between the CONUT and PNI score at admission and long-term adverse outcomes in adults with AIS. Overall, the results of this study suggest that AIS patients with impaired immune-nutritional status are at a significantly increased risk of developing RIS and MACEs in the period beyond three years after AIS onset.

Malnutrition, which was assessed by the PNI or CONUT, was associated with a higher risk of mortality and MACEs in studies of heart failure and acute cardiovascular diseases [10,22,23]. Previous studies which investigated the predictive value of PNI and CONUT in AIS patients were mainly focused on short-term prognoses [3,12,24,25], while their prognostic significance for long-term outcomes remained unclear. Zhang et al. have reported that malnutrition at admission may predict 12-month functional recovery in AIS patients [7]. Yuan et al. have reported an association between undernourishment and long-term mortality in the elderly with a first ischemic stroke, applying the CONUT and PNI [11]. Moreover, moderate malnutrition risk according to PNI score was associated with long-term incident ischemic stroke risk in patients with acute coronary syndrome (ACS) [10]. The findings of our study supported that PNI and CONUT indices have the potential to identify AIS patients not only with increased mortality risk but also with increased risk of RIS and MACEs in long-term follow-up. The result of the present study was compatible with Zhang et al.’s findings that undernutrition assessed at admission may predict 12-month outcomes in AIS patients [7].

Although both malnutritional indices were associated with an unfavorable long-term prediction for RIS and MACEs, the CONUT score exhibited a negative linear association with poor prognosis in the present study, whereas the PNI score showed a nonlinear association (*p* for linearity < 0.05). Consistent with the previous studies, the higher CONUT score, which means a worsened nutrition status, predicted adverse cardio-cerebrovascular events. In addition, we established a U-shaped relationship between PNI and RIS, and a PNI value at 46.18 as the lowest risk point for poor outcomes in the present study. Furthermore, the cutoff value around 44.75 was confirmed by multivariable regression analysis after classing the PNI into tertiles. Therefore, when the PNI score was treated as a binary variable (low PNI score (≤44.75) and high PNI score (>44.75)), it was more predictive than its standard scoring system (absent, moderate, and severe) in the AIS population. Our finding was comparable with the study that investigated the long-term prospective implications of malnutrition in carotid artery stenting (CAS) patients, where results have indicated that at a cutoff value of 1.5 and 46, respectively, the CONUT and PNI score predicted long-term all-cause death and stroke with moderate sensitivity and a specificity [26]. 

Inflammation and oxidative stress play a critical role in stroke pathogenesis. The indices’ constituents might account for the association between the two malnutrition indices and RIS. In ischemic stroke, serum albumin, a multifunctional protein, exhibits neuroprotective properties, such as preventing erythrocyte aggregation [27], and posing as a major antioxidant [28]. Low albumin level was related significantly to poor outcomes among all stroke subtypes [29,30] and increased risk of recurrence in patients with AIS. The immune response implicated in the pathogenesis of ischemic stroke is complicated [31,32]. In the inflammatory process, lymphocytes can infiltrate ischemic regions after AIS [33]. Acutely, lymphocytes may result in the release of pro-inflammatory cytokines and cytotoxic substances, which have a detrimental effect [34]. Chronically, reports suggested that lymphocytes are also indispensable for tissue repairing and remodeling [35,36]. Higher lymphocyte counts at admission were associated with a decreased risk of death, stroke recurrence, and poor neurological prognosis at one-year follow-up after AIS in a Chinese cohort study [37]. In the present study, lymphocyte counts were significantly lower in the group of patients with RIS, which is consistent with previous reports.

The relationship between total cholesterol (TC) and stroke has been inconsistent. Low TC levels in stroke patients may act as a double-edged sword, lowering the risk of ischemic stroke while increasing the risk of hemorrhagic stroke [38,39]. Additionally, Zhou, et al. have reported that patients with atherosclerotic infarction and low cholesterol levels treated with statins had increased long-term dependency and recurrence risk after AIS [40]. TC may represent the individual nutritional status to some extent and a non-linear relationship may correlate with adverse outcomes in AIS patients [39]. TC was generally accepted as related to atherosclerosis. The presence of atherosclerosis can cause malnutrition and on another hand, the presence of malnutrition may be one of the risk factors for developing atherosclerosis [41]. The chronic inflammatory response of leukocytes in the arterial wall results in the formation of intracranial and extracranial carotid plaques [42]. Nutritional deficiencies are associated with compromised immune function, which translates into an increased burden of atherosclerosis [10]. In the subgroup analysis, malnutrition according to CONUT particularly associated with RIS and MACEs in subgroup patients with large arteriosclerosis etiology, suggesting the interactional relationship between malnutrition and atherosclerosis.

Detailed assessment of a patient’s nutritional status has always been considered difficult due to the time-critical nature of the stroke process. Currently, there is no consensus on which malnutrition screening instrument to use in patients with AIS. CONUT or PNI scores may better reflect the balance of the nutrition and inflammation of the subject than single markers. The present study provided further evidence that objective malnutritional indices improve the prediction of risk classification for long-term adverse outcomes, as validated by reclassification statistics and decision curve analysis.

The present study has several limitations. First, this was a retrospective study conducted at a single center in China, with a possible selection bias. However, the baseline characteristics of participants were not significantly different from those reported in a previous large stroke registry study in China [43]. Second, owing to the study’s retrospective nature, the CONUT and PNI scores were not assessed after discharge, and thus we did not evaluate the effect of the longitudinal change of malnutritional indices on the prognosis during the follow-up period. Finally, the validity of nutritional status assessed by the CONUT or PNI is unconfirmed due to the absence of comparison with comprehensive nutritional assessments, such as the Nutritional Risk Screening 2002 and Malnutrition Universal Screening Tool. Confirmation of our result by other investigators and in other populations is recommended. Authors should discuss the results and how they can be interpreted from the perspective of previous studies and of the working hypotheses. The findings and their implications should be discussed in the broadest context possible. Future research directions may also be highlighted.

## 5. Conclusions

In conclusion, our study revealed that malnutrition at admission in patients with adult ischemic stroke is associated with a greater risk of future RIS and MACEs. The CONUT and PNI could be useful indicators of immune-nutritional state for predicting outcomes and facilitating prognostic improvement in AIS patients by determining those who might benefit from nutritional intervention. Further studies are warranted to assess the effectiveness of nutritional management in patients suffering AIS based on the two indicators.

## Figures and Tables

**Table 1:** Details of the two malnutrition scoring systems.

Nutritional Scores			Risk of Malnutrition	
	Absent	Mild	Moderate	Severe
CONUT, points	0–1	2–4	5–8	9–12
Albumin, g/L	≥35	30–34.9	25–29.9	<25
Score	0	2	4	6
Total cholesterol, mg/dL	≥180	140–179	100–139	<100
Score	0	1	2	3
Lymphocyte count, ×10^9^/L	≥1.60	1.20–1.59	0.80–1.19	<0.80
Score	0	1	2	3
PNI, points	>38		35–38	<35
Formula: 5 × lymphocyte count (10^9^/L) + serum albumin concentration (g/L)				

Abbreviations: CONUT, controlling nutritional status score; PNI, prognostic nutritional index.

**Table 2:** Baseline data of patients with and without recurrent ischemic stroke.

Variables	Total (*n* = 991)	Non-RIS (*n* = 788)	RIS (*n* = 203)	*p*
Age, Median (IQR)	66 (58, 74)	65 (56, 73)	70 (62.5, 76)	<0.001 *
Sex, Male *n* (%)	699 (71)	550 (70)	149 (73)	0.359
DM, *n* (%)	185 (19)	152 (19)	33 (16)	0.375
HTN, *n* (%)	526 (53)	403 (51)	123 (61)	0.02 *
IS, *n* (%)	229 (23)	169 (21)	60 (30)	0.019 *
ICH, *n* (%)	20 (2)	13 (2)	7 (3)	0.155
SBP, Median (IQR), mmHg	148 (135, 163)	149 (135, 164)	146 (132.5, 160)	0.059
DBP, Median (IQR), mmHg	85 (76, 93)	85 (76, 94)	83 (75, 92)	0.163
WBC, Median (IQR), ×10^9^/L	7.97 (6.47, 9.98)	8 (6.58, 10)	7.82 (6.04, 9.8)	0.218
RBC, Median (IQR), ×10^12^/L	4.58 (4.23, 4.97)	4.59 (4.25, 5)	4.48 (4.2, 4.86)	0.022 *
LYM, Median (IQR), ×10^9^/l	1.68 (1.29, 2.18)	1.72 (1.33, 2.23)	1.5 (1.19, 2.04)	<0.001 *
ALT, Median (IQR), μ/L	16 (12, 23)	16 (12, 22)	17 (12, 24)	0.344
ALB, Median (IQR), g/L	38.1 (35.6, 40.5)	38.45 (36, 40.7)	36.4 (33.9, 39.5)	<0.001 *
Scr, Median (IQR), μmol/L	76 (58.1, 93.2)	75 (57.85, 92.23)	79.8 (59.8, 100.5)	0.025 *
FBS, Median (IQR), mmol/L	5 (5, 6)	5 (5, 6)	5 (5, 6)	0.257
TC, Median (IQR), mg/dL	182.09 (154.64, 213.4)	183.25 (155.03, 214.56)	178.22 (151.55, 207.6)	0.319
TOAST, *n* (%)				0.024 *
LAA	454 (46)	380 (48)	74 (36)	
CE	138 (14)	101 (13)	37 (18)	
SAA	336 (34)	256 (32)	80 (39)	
SOE	25 (3)	19 (2)	6 (3)	
SUE	38 (4)	32 (4)	6 (3)	
NIHSS at admission, Median (IQR)	3 (2, 5)	3 (2, 5)	3 (2, 5)	0.748
Premorbid mRS, Median (IQR)	0 (0, 0)	0 (0, 0)	0 (0, 0)	0.076
ND, *n* (%)	123 (12)	111 (14)	12 (6)	0.002 *
CONUT, Median (IQR)	2 (0, 3)	1 (0, 3)	2 (1, 3)	<0.001 *
CONUT scoring system, *n* (%)				<0.001 *
absent	485 (49)	407 (52)	78 (38)	
mild	445 (45)	350 (44)	95 (47)	
moderate	56 (6)	29 (4)	27 (13)	
severe	5 (1)	2 (0)	3 (1)	
PNI, Median (IQR)	46.3(43.55, 50.15)	46.65 (44.45, 50.35)	43.7 (40.48, 49.1)	<0.001 *
PNI scoring system, *n* (%)				<0.001 *
absent	952 (96)	767 (97)	185 (91)	
moderate	26 (3)	14 (2)	12 (6)	
severe	13 (1)	7 (1)	6 (3)	

Abbreviations: RIS, recurrent ischemic stroke; IQR, interquartile range; DM, diabetes mellitus; HTN, hypertension; IS, ischemic stroke; ICH, intracerebral hemorrhage; SBP, systolic blood pressure; DBP, diastolic blood pressure; WBC, white blood cell; RBC, red blood cell; LYM, lymphocyte; PLT, platelet; ALT, alanine transaminase; ALB, albumin; Scr, serum creatinine; FBS, fasting blood sugar; TC, total cholesterol; TOAST, the Trial of ORG 10172 in Acute Stroke Treatment; large-artery atherosclerosis; CE, cardioembolism; SAA, small-vessel occlusion; SOE, stroke of other determined etiology; SUE, stroke of undetermined etiology; NIHSS, National Institutes of Health Stroke Scale; mRS, modified Rankin Scale; ND, neurological deterioration; CONUT, Controlling Nutritional Status score; PNI, Prognostic Nutritional Index. * *p* < 0.05.

**Table 3:** Multivariable analysis of two malnutrition indexes to predict recurrent ischemic stroke.

	Model 1 ^†^		Model 2 ^‡^		Model 3 ^§^	
Index	AdjustedHR (95%CI)	*p*	AdjustedHR (95%CI)	*p*	AdjustedHR(95%CI)	*p*
PNI categories						
Tertile 1 (≤44.75)	1.0 [Reference]		1.0 [Reference]		1.0 [Reference]	
Tertile 2 (44.76–48.9)	0.293 (0.201–0.427)	<0.001	0.290 (0.199–0.423)	<0.001	0.295 (0.202–0.430)	<0.001
Tertile 3 (>48.9)	0.446 (0.314–0.633)	<0.001	0.439 (0.307–0.629)	<0.001	0.445 (0.308–0.632)	<0.001
PNI as bivariate (≤44.75)	2.627 (1.610–4.289)	<0.001	2.733 (1.547–4.536)	<0.001	2.782 (2.073–3.730)	<0.001
PNI per 1-point increase	0.927 (0.901–0.952)	<0.001	0.920 (0.895–0.949)	<0.001	0.922 (0.8963–0.948)	<0.001
CONUT categories						
Normal	1.0 [Reference]		1.0 [Reference]		1.0 [Reference]	
Mild	1.246 (0.916–1.694)	0.161	1.234 (0.904–1.685)	0.183	1.224 (0.898–1.668)	0.200
Moderate-severe	3.551 (2.304–5.470)	<0.001	3.563 (2.276–5.576)	<0.001	3.472 (2.223–5.423)	<0.001
CONUT as bivariate (>1)	1.456 (1.088–1.949)	0.012	1.432 (1.066–1.925)	0.017	1.443 (1.081–1.943)	0.012
CONUT per 1-point increase	1.195 (1.112–1.284)	<0.001	1.200 (1.112–1.296)	<0.001	1.206 (1.117–1.301)	<0.001

† Model 1, adjusted for age, sex. ‡ Model 2, adjusted for age, sex, stroke etiology, smoking status, history of ischemic stroke, history of diabetes mellitus, history of hypertension, and NIHSS score at admission. § Model 3, adjusted for age, serum creatinine, stroke etiology, history of ischemic stroke, history of hypertension, platelet count, and neurological deterioration.

**Table 4:** Reclassification statistics (95% CI) for RIS and MACEs after the addition of two malnutrition indices.

Model	C-Index	cNRI	*p*-Value	IDI	*p*-Value
RIS					
Model 3 ^†^	0.633	Reference		Reference	
Model 3 + PNI	0.673	0.219 (0.119–0.315)	0.002	0.028 (0.009–0.059)	<0.001
Model 3 + CONUT	0.661	0.164 (0.071–0.244)	<0.001	0.019 (0.003–0.045)	0.004
MACEs					
Model 3	0.638	Reference		Reference	
Model 3 + PNI	0.673	0.208 (0.120–0.295)	<0.001	0.032 (0.012–0.059)	<0.001
Model 3 + CONUT	0.666	0.183 (0.098–0.246)	<0.001	0.024 (0.007–0.050)	<0.001

Abbreviations: cNRI, continuous reclassification improvement; IDI, integrated discrimination improvement; RIS, recurrent ischemic stroke; MACEs, major cardiovascular events; CONUT, controlling nutritional status score; PNI, prognostic nutritional index. † Model 3, adjusted for age, serum creatinine, stroke etiology, history of ischemic stroke, history of hypertension, platelet count, neurological deterioration.

## Data Availability

The data used and analyzed during the current study are available from the corresponding author on reasonable request.

## Supplementary Materials

The following supporting information can be reviewed at: https://www.mdpi.com/article/10.3390/nu14071337/s1. Supplementary Materials Zip, Table S1: Univariate Cox regression analysis according to patients with and without recurrent ischemic stroke; Table S2: Multivariate analysis of two malnutrition indexes to predict major cardiovascular events.
