# Supplementary material for: Baseline Objective Malnutritional Indices as Immune-Nutritional Predictors of Long-Term Recurrence in Patients with Acute Ischemic Stroke
Source: Nutrients. 2022 Mar 23;14(7):1337. doi: 10.3390/nu14071337 (PMC9000876; doi:10.3390/nu14071337)
Supplement: Supplementary file 1 [file nutrients-14-01337-s001.zip › Supplementary Table S2.pdf]

Supplementary Table S2. Multivariate analysis of two malnutrition indexes to predict major cardiovascular events.

	Model 1†		Model 2‡		Model3§	
Index	adjustedHR (95%CI)	*P*	adjustedHR(95%CI)	*P*	adjustedHR(95%CI)	*P*
PNI categories						
Tertile1(≤44.75)	1.0 [Reference]		1.0 [Reference]		1.0 [Reference]	
Tertile2(44.76-48.9)	0.320(0.231-0.442)	**<0.001**	0.318(0.230-0.441)	**<0.001**	0.232(0.233-0.447)	**<0.001**
Tertile3(> 48.9)	0.449(0.329-0.614)	**<0.001**	0.443(0.322-0.610)	**<0.001**	0.450(0.327-0.618)	**<0.001**
PNI per 1-point increase	0.931(0.908-0.953)	**<0.001**	0.927(0.904-0.951)	**<0.001**	0.928(0.905-0.952)	**<0.001**
PNI as bivariate (≤ 44.75)	2.646(2.045-3.424)	**<0.001**	2.679(2.0618-3.479)	**<0.001**	2.642 (2.033-3.434)	**<0.001**
CONUT categories						
Normal	1.0 [Reference]		1.0 [Reference]		1.0 [Reference]	
Mild	1.388(1.058-1.821)	**0.017**	1.406(1.066-1.845)	**0.015**	1.352(1.029-1.777)	**0.03**
Moderate-severe	3.483(2.356-5.150)	**<0.001**	3.359(2.238-5.041)	**<0.001**	3.288(2.195-4.927)	**<0.001**
CONUT as bivariate (> 1)	1.580(1.129-2.048)	**<0.001**	1.577(1.212-2.505)	**<0.001**	1.513 (1.163-1.967)	**0.002**
CONUT per 1-point increase	1.193(1.119-1.270)	**<0.001**	1.194(1.116-1.276)	**<0.001**	1.190(1.115-1.275)	**<0.001**

†Model 1, adjusted for age, sex.

‡Model 2, adjusted for age, sex, stroke etiology, smoking status, and history of ischemic stroke, history of diabetes mellitus, history of hypertension, NIHSS at admission.

§Model 3, adjusted for age, serum creatinine, stroke etiology, history of ischemic stroke, history of hypertension, platelet count, neurological deterioration.
