# Supplementary material for: Baseline Objective Malnutritional Indices as Immune-Nutritional Predictors of Long-Term Recurrence in Patients with Acute Ischemic Stroke
Source: Nutrients. 2022 Mar 23;14(7):1337. doi: 10.3390/nu14071337 (PMC9000876; doi:10.3390/nu14071337)
Supplement: Supplementary file 1 [file nutrients-14-01337-s001.zip › Supplementary Table S1.pdf]

Table S1. Univariate Cox regression analysis according to patients with and without recurrent ischemic stroke.

Variable	HR	95%CI	*p*	Variable	HR	95%CI	*p*
Age	1.03	1.02-1.05	**<0.001**	LDL, mmol/l	0.885	0.763-1.03	0.108
Sex(male)	1.14	0.839-1.56	0.394	Triglyceric, mmol/l	0.916	0.802-1.05	0.200
Diabetes mellitus	0.86	0.592-1.25	0.428	TOAST			*0.043*
Hypertension	1.45	1.09-1.92	**0.009**	LAA	reference		
Ischemic Stroke	1.5	1.11-2.03	**0.008**	CE	1.8	1.21-2.67	**0.003**
Cerebral hemorrhage	1.66	0.78-3.52	0.189	SAA	1.35	0.984-1.86	0.062
Smoke status	1.09	0.823-1.43	0.563	SOE	1.34	0.582-3.08	0.492
Baseline SBP, mmHg	0.995	0.989-1	0.111	SUE	0.85	0.37-1.96	0.702
Baseline DBP, mmHg	0.993	0.983-1	0.226	NIHSS at admission	1.02	0.979-1.06	0.382
WBC, ×10^9/l	0.981	0.933-1.03	0.468	ND	0.462	0.258-0.829	**0.009**
				Premorbid mRS	1.208	0.975-1.408	0.083
NEU, ×10^9/l	1	0.955-1.06	0.857	Malnutrition index			
LYM, ×10^9/l	0.689	0.556-0.853	**<0.001**	CONUT score	1.24	1.16-1.32	**<0.001**
RBC, ×10^{12}/l	1	0.994-1.01	0.851	PNI score	0.917	0.893-0.94	**<0.001**
Hb,g/l	0.996	0.988-1	0.360	CONUT categories			**<0.001**
PLT, ×109/l	0.998	0.996-1	0.047	absent	reference		
ALT, u/l	1	0.996-1.01	0.301	mild	1.44	1.07-1.94	**0.017**
ALB, g/l	0.912	0.883-0.941	**<0.001**	moderate-severe	4.21	2.75-6.43	**<0.001**
Scr,μmol/l	1	1-1.01	**0.014**	PNI categories			**<0.001**
FBS, mmol/l	0.996	0.94-1.06	0.890	tertile1(≤44.75）	reference		
TC, mg/dl	0.931	0.826-1.05	0.245	tertile2(44.76-48.90）	0.266	0.184-0.384	**<0.001**
HDL, mmol/l	1.17	0.758-1.8	0.480	tertile3(>48.90）	0.371	0.267-0.515	**<0.001**

Abbreviations: RIS, recurrent ischemic stroke; IQR, interquartile ranges; DM, diabetes mellitus; HTN, hypertension; IS, ischemic stroke; ICH, intracerebral hemorrhage; SBP, systolic blood pressure; DBP, diastolic blood pressure; WBC, white blood cell; NEU, leukocyte; LYM, lymphocyte; Hb, hemoglobin; RBC, red blood cell; PLT, platelet; ALT, alanine transaminase; ALB, albumin; Scr, serum creatinine; FBS, fasting blood sugar; TC, total cholesterol; HDL, high density lipoprotein; LDL, low density lipoprotein; TOAST, the trial of ORG 10172 in Acute Stroke Treatment; large-artery atherosclerosis; CE, cardioembolism; SAA, small-vessel occlusion; SOE, stroke of other determined etiology; SUE, stroke of undetermined etiology; NIHSS, National Institute of Health Stroke Scale; ND, neurological deterioration; mRS, modified Rankin Scale; CONUT, controlling nutritional status score; PNI, prognostic nutritional index.
